# Profile of the Health Planning Management Workforce: a cross-sectional study, Rio Grande do Sul, Brazil, 2023

**DOI:** 10.1590/S2237-96222026v35e20250590.en

**Published:** 2026-04-04

**Authors:** Caroline Cardozo Bortolotto, Pedro San Martin Soares, André Luis Alves de Quevedo, Giuliani Schwantz, Isana Oliveira da Silva, Cristian Fabiano Guimaraes

**Affiliations:** 1 Secretaria da Saúde do Estado do Rio Grande do Sul, Porto Alegre, RS, Brazil Secretaria da Saúde do Estado do Rio Grande do Sul Porto Alegre RS Brazil; 2 Tribunal de Contas do Estado do Rio Grande do Sul, Porto Alegre, RS, Brazil Tribunal de Contas do Estado do Rio Grande do Sul Porto Alegre RS Brazil; 3 Universidade Federal de São Paulo, Departamento de Medicina Preventiva da Escola Paulista de Medicina, São Paulo, SP, Brazil Universidade Federal de São Paulo, Departamento de Medicina Preventiva da Escola Paulista de Medicina São Paulo SP Brazil

**Keywords:** Health Planning, Health Personnel, Health Manager, Health Management, Health Services, Planificación en Salud, Personal de Salud, Gestor de Salud, Gestión en Salud, Servicios de Salud.

## Abstract

**Objective:**

To describe the profile of the workforce involved in health planning management within the Brazilian National Health System (SUS) at the level of municipal health departments in the state of Rio Grande do Sul, Brazil.

**Methods:**

This was a cross-sectional study conducted with municipal health secretaries and health planning management workers through self-administered online questionnaires, carried out between September and November 2023. Descriptive analysis and association tests were performed, considering statistical significance for p-value<0.05 using Pearson's chi-square test.

**Results:**

Of the 497 municipalities in the state, 467 municipal health secretaries and 431 SUS planning management workers participated in the study. Most participants were female, over 40 years old, and self-identified as White. Workers reported a higher level of education than municipal health secretaries. In terms of their employment status with the municipal executive branch, most secretaries held appointed positions (74.08%), while most workers were public servants (60.32%). Approximately 68% of health workers reported not working exclusively in municipal health planning management. The larger the population size of the municipality, the greater the likelihood of having dedicated health planning staff and a specific department for SUS planning management.

**Conclusion:**

Understanding who the actors involved in SUS planning and management are may support public policy analysis and the adoption of strategies aimed at improving the quality of the system and the health status of the population.

## Resumen

**Objetivo:**

Describir el perfil del personal involucrado en la gestión de la planificación en salud en el marco del Sistema Único de Salud (SUS) de Brasil, en las secretarías municipales de salud del estado de Rio Grande do Sul.

**Métodos:**

Estudio transversal realizado con secretarios municipales de salud y trabajadores de la gestión de la planificación en salud, mediante cuestionarios en línea autoadministrados, aplicados entre septiembre y noviembre de 2023. Se realizaron análisis descriptivos y pruebas de asociación, considerando significancia estadística para p-valor<0,05 utilizando la prueba chi-cuadrado de Pearson.

**Resultados:**

De los 497 municipios del estado, participaron en el estudio 467 secretarios municipales de salud y 431 trabajadores de la gestión de la planificación del SUS. La mayoría de los participantes eran mujeres, mayores de 40 años, y se autodeclararon de raza/cor blanca. Los trabajadores reportaron un mayor nivel educativo que los secretarios municipales de salud. En cuanto al vínculo laboral con el poder ejecutivo municipal, la mayoría de los secretarios ocupaban cargos comisionados (74,08%), mientras que la mayoría de los trabajadores eran funcionarios públicos (60,32%). Aproximadamente el 68% de los trabajadores de salud declararon no trabajar exclusivamente en la gestión de la planificación en salud en el ámbito municipal. A mayor tamaño poblacional del municipio, mayor fue la probabilidad de contar con personal exclusivo para la planificación en salud y con un sector específico para la gestión de la planificación del SUS.

**Conclusión:**

Conocer quiénes son los actores involucrados en la planificación y gestión del SUS puede apoyar el análisis de políticas públicas y la adopción de estrategias orientadas a mejorar la calidad del sistema y la situación de salud de la población.

**Ethical aspects**

This research respected ethical principles, having obtained the following approval data:

Research ethics committee: Escola de Saúde Pública do Rio Grande do Sul

Opinion number: 6277600

Approval date: 1/9/2023

Certificate of submission for ethical appraisal: 69138023,9,0000,5312

Informed consent record: Obtained from all participants.

## Introduction 

In Brazil, government planning in its contemporary form was institutionalized mainly from 1990 onward. According to Law No. 8,080, of September 19, 1990 ^1^, which created the Brazilian National Health System (SUS), the national management of the system is responsible for developing the country's strategic plan, in technical cooperation with the states, municipalities, and the Federal District, emphasising a bottom-up logic in the planning process. Therefore, adequate planning depends on robust structures and a consistent workforce at the state and municipal levels.

In this article, the health planning management workforce comprises municipal health secretaries and workers within the area of Brazilian public administration. The former, appointed by the democratically elected government, are primarily responsible for decision-making in management processes; the latter are tasked with technical and political structuring and implementation of actions in this field.

SUS is a complex system that requires efficient planning to ensure the provision of quality health services to the population. Accordingly, planning is understood to be an instrument for agreeing on the responsibilities of the three spheres of government, which, in addition to being a legal requirement, allows for the definition of objectives and the organization of actions to be implemented. Within this framework, planning is articulated through intentionally designed mechanisms and methodologies, alongside work processes that require specialized technical and managerial knowledge ^2^^-^^5^.

Managing health presents countless challenges in terms of decision-making, and planning is a necessary function to provide support in the coordination inherent to this complex and dynamic process. Nevertheless, despite there being a consensus that planning is a strategic tool for designing public health policy in Brazil, health planning has rarely been considered as a strategy and policy with a view to analyzing its strengths and challenges ^2^^-^^3^. 

From the 2000s onwards, there has been a strong incentive within SUS for health management and planning processes at the state and municipal health department levels. Through the SUS Planning System, it was possible to establish a policy for the systematic implementation of regulatory management instruments, such as health insurance plans, annual health programs, and management reports ^4^^-^^6^. The purpose of this policy is to improve the practice of professionals working in this field.

Despite improvements in this field, few studies in academic literature have evaluated the profile of secretaries and municipal health workers involved in SUS planning management ^2^^,^^5^^,^^7^^-^^10^, which results in a gap in knowledge and in processes to enhance health management and planning. Thus, this article aims to describe the profile of the workforce involved in planning management for the Brazilian National Health System within the municipal health departments of Rio Grande do Sul. 

## Methods 

Study Design

This cross-sectional study was conducted in Rio Grande do Sul with 467 municipal health secretaries and 431 municipal health workers responsible for managing the planning of the Brazilian National Health System (SUS) in the municipalities of the state.

Setting

Rio Grande do Sul comprises 497 municipalities and a population of 10,882,965 people, according to the 2022 Brazilian census ^11^. Regarding population size, 335 municipalities have up to 10,000 inhabitants; 55 have between 10,000 and 20,000; 64 between 20,000 and 50,000; 24 between 50,000 and 100,000; and 19 municipalities have more than 100,000 inhabitants ^8^. Territorially, the health system is organized into seven health macro-regions (Central-West, Metropolitan, Missioneira, North, Serra, South, and Vales), 18 regional health coordinators, and 30 health regions ^12^.

Participants

All individuals over 18 years of age who were municipal health secretaries or workers responsible for planning the Brazilian National Health System (SUS) in the municipal health departments of Rio Grande do Sul were eligible to participate in the study. As for attempts to increase participation, health management spaces were used, such as regional interagency committees, institutional working groups, contacts via the Rio Grande do Sul State Court of Auditors, and the Rio Grande do Sul State Council of Municipal Health Secretariats, in addition to sending letters to mayors explaining the relevance of the study.

Variables

The variables selected for this article were: sex (male; female); age (in years); race/skin color (White; Black; Brown; other); educational level (incomplete/complete elementary education; incomplete/complete secondary education; incomplete higher education; complete higher education; specialization degree; Master's/Doctoral degree); employment relationship with the municipal executive branch (public servant; appointed position; CLT employee (employee under the Brazilian Consolidation of Labor Laws - CLT); fixed-term contract; outsourced/service provider); undergraduate field (list of professions in the health area, human sciences, and applied social sciences, plus an open field); postgraduate studies in management or public health planning (yes; no); experience in SUS planning management before becoming municipal health secretary (yes; no); previous experience in SUS planning management (in years); length of service at the municipal health department (in years); exclusively working with SUS planning management in the municipality (yes; no); existence of a specific sector for SUS planning management within the municipal health department (yes; no); whether workers responsible for entering information into the Brazilian Health Budget Information System had an employment relationship with the municipal health department (yes; no); whether the municipal health fund manager was the municipal health secretary (yes; no); population size (<10,000 inhabitants; 10,000 to <20,000; 20,000 to <50,000; 50,000 to <100,000; ≥100,000 inhabitants).

Source, data measurement, and bias control

The two data collection instruments (one for secretaries and one for municipal SUS planning management workers) were sent via the internal system of the State Court of Auditors of Rio Grande do Sul. Both were self-administered online questionnaires, developed based on the researchers' previous experience and on a study conducted in the state of Santa Catarina, which aimed to evaluate health planning management in municipalities of that state ^2^.

Initially, a pilot study was conducted to test the data collection instruments and assess the feasibility and effectiveness of the proposed study methodology. For this purpose, three health macro-regions of Rio Grande do Sul (Central-West, Missioneira, and Vales) were randomly selected and, subsequently, three municipalities (Silveira Martins, Boa Vista do Incra, and Herveira) with fewer than 3,000 inhabitants were randomly selected-one in each of the selected health macro-regions. Analyses were performed to assess the consistency of responses and the quality of the data obtained. After this process, the study was expanded to the remaining municipalities of Rio Grande do Sul. The municipalities included in the pilot study were not considered in the final analysis of this study.

Regarding the recruitment and selection of participants, first, a presentation letter about the study was sent by email to municipal health secretaries. Based on the presentation letter, the municipal secretary informed the workers responsible for managing the SUS planning in the municipality about the study and that they would be contacted in the future to be asked to complete the questionnaire. 

Data collection took place between September and November 2023 and was supervised by two epidemiologists. Data consistency checks were performed weekly. Finally, the bias control strategies employed included: defining inclusion and exclusion criteria; supervision of data collection; consistency checks of responses; loss analyses; and stratification of the results.

Statistical analysis

This study did not apply sample size calculation, as the research aimed to include the entire universe of municipal health departments in the form of a municipal census of SUS health planning management. Data analysis was performed using the Stata 17.0 statistical package. Initially, descriptive analyses of the variables were conducted, with categorical variables presented as absolute and relative frequencies. For symmetrically distributed data, means and standard deviations (SD) were calculated, while for asymmetrically distributed data, medians and interquartile ranges (IQR) were used. To assess associations, Pearson's chi-square test was applied, considering p-values<0.05 as statistically significant.

## Results 

Among the 497 municipalities, responses were obtained from 467 (93.96%) municipal health secretaries and 431 (86.72%) municipal health workers involved in health planning. Most respondents were female (52.68% of secretaries and 77.26% of workers). Age distribution was concentrated between 30 and 59 years. The proportion of individuals aged 60 years or older was higher among secretaries than among workers (12.21% and 3.48%, respectively). Additionally, 93.27% of respondents self-identified as White (Table 1). The average age was 45.96 years for secretaries (95%CI 45.05-46.87; SD 10.05) and 41.28 years for workers (95%CI 40.45-42.10; SD 8.70). 

Regarding educational level, 37.47% of secretaries had not completed higher education; 26.98% of secretaries and 43.39% of workers held a specialization degree. Most secretaries were employed in commissioned positions within the municipal executive branch (74.08%), while most workers were public servants (60.32%) (Table 1).

Among secretaries with higher education (n=292), the main fields of study were: nursing (n=62), administration (n=46), law (n=24), health management (n=20), pedagogy (n=14), social work (n=13), and other fields (n=113). Among workers with higher education (n=367), the main fields of study were: nursing (n=180), administration (n=33), law (n=17), accounting (n=16), social work (n=8), dentistry (n=8), and other fields (n=105).

Of the 292 secretaries with higher education, 29.45% (n=86) reported having a postgraduate degree in health management or planning. This proportion was 33.24% among workers. With regard to prior experience in SUS health planning management, 37.05% (n=179) of secretaries reported having such experience before assuming their current position. The median time of previous experience in SUS planning management among these secretaries was 2.75 years (IQR 0.58-6.67 years).

**Table 1:** Characteristics of municipal health secretaries (n=467) and workers (n=431) involved in planning management for the Brazilian National Health System. Rio Grande do Sul, 2023

Variable	Secretaries n (%)	Workers n (%)
Sex		
Female	246 (52.68)	333 (77.26)
Male	221 (47.32)	98 (22.74)
Age group (years)		
18-29	12 ( 2.57)	35 (8.12)
30-39	119 (25.48)	151 (35.03)
40-49	176 (37.69)	178 (41.30)
50-59	103 (22.06)	52 (12.06)
60≥	57 (12.21)	15 (3.48)
Race/skin color		
White	437 (93.58)	402 (93.27)
Black	25 (5.35)	19 (4.41)
Brown/Other	5 (1.07)	10 (2.31)
Education level		
Incomplete/complete elementary school	19 (4.07)	2 (0.46)
Incomplete/complete high school	107 (22.91)	39 (9.05)
Incomplete higher education	49 (10.49)	23 (5.34)
Complete higher education	149 (31.91)	163 (37.82)
Specialist degree	126 (26.98)	187 (43.39)
Master's/Doctoral degree	17 (3.64)	17 (3.94)
Employment relationship with the municipal executive branch		
Public servant	108 (23.13)	260 (60.32)
Appointed position	346 (74.08)	114 (26.45)
CLT employee	4 (0.86)	9 (2.09)
Fixed-term contract	7 (1.50)	28 (6.50)
Outsourced worker/service provider	2 (0.43)	20 (4.64)

The median length of service in the municipal health department was 2.75 years (IQR 0.83-6.25 years) for secretaries and 4.8 years (IQR 1.7-13 years) for workers. When stratified by length of service, 71.73% of secretaries and 51.51% of workers had been employed for less than five years. Additionally, 11.56% of secretaries and 22.97% of workers reported having been working for 10 to 20 years. There was no statistically significant association between length of employment and municipal population size.

Approximately 68% of health workers reported not working exclusively with health planning at the municipal level. When stratified by population size, municipalities with fewer than 10,000 inhabitants showed a 31.3 percentage point higher rate of respondents not working exclusively with planning compared to municipalities with 100,000 inhabitants or more (72.50% vs. 41.20%; p=0.027) (Table 2). 

More than half of secretaries (50.96%) and workers (51.28%) reported the existence of a dedicated sector for SUS planning within the municipal health department. Moreover, municipalities with 100,000 inhabitants or more had a higher prevalence of having a specific sector for SUS planning (84.21%; p=0.025 among secretaries; 88.24%; p=0.006 among workers) (Table 3).

When asked whether those responsible for entering data into the Brazilian Health Budget Information System had an employment relationship with the municipal health department, only 177 (37.90%) secretaries responded affirmatively. It was found that the larger the population size of the municipalities, the higher the prevalence of workers employed by the health department to enter information into the Brazilian Health Budget Information System (p-value<0.001) (Table 4). Lastly, 88.15% of municipal secretaries reported being managers of the Municipal Health Fund. When this variable was stratified by population size, there was no statistical significance. 

**Table 2:** Exclusively working in the management of the Brazilian National Health System (SUS) planning, by population size, municipal health workers in SUS planning management. Rio Grande do Sul, 2023 (n=431)

Exclusively working in SUS planning management (p-value 0,027)^a^	<10,000 inhabitants	10,000 to <20,000	20,000 to <50,000	50,000 to <100,000	≥100,000 inhabitants
	n(%)	n(%)	n(%)	n(%)	n(%)
No	206 (72.50)	32 (62.75)	33 (56.90)	13 (61.90)	7 (41.20)
Yes	78 (27.50)	19 (37.25)	25 (43.10)	8 (38.10)	10 (58.80)

a Pearson's chi-square test for heterogeneity.

**Table 3:** Existence of a specific sector for the Brazilian National Health System (SUS) planning, by population size, municipal health secretaries (n=467), and municipal health workers (n=431) in SUS planning management. Rio Grande do Sul, 2023

Specific sector for SUS planning management	<10,000 inhabitants	10,000 to <20,000	20,000 to <50,000	50,000 to <100,000	≥100,000 inhabitants
	n (%)	n (%)	n (%)	n (%)	n (%)
Secretaries (p-value 0,025)^a^					
No	161 (51.77)	29 (53.70)	28 (45.16)	8 (38.10)	3 (15.79)
Yes	150 (48.23)	25 (46.30)	34 (54.84)	13 (61.90)	16 (84.21)
Workers (p-value 0,006)^a^					
No	153 (53.87)	22 (43.14)	25 (43.10)	8 (38.10)	2 (11.76)
Yes	131 (46.13)	29 (56.86)	33 (56.90)	13 (61.90)	15 (88.24)

a Pearson's chi-square test for heterogeneity.

**Table 4:** Employment relationship of workers responsible for entering information into the Brazilian Health Budget Information System, by population size, municipal health secretaries in the Brazilian National Health System planning management. Rio Grande do Sul, 2023 (n=467)

Employment relationship of health worker (p-value<0,001)^a^	<10,000 inhabitants	10,000 to <20,000	20,000 to <50,000	50,000 to <100,000	≥100,000 inhabitants
	n (%)	n (%)	n (%)	n (%)	n (%)
No	218 (70.32)	34 (62.96)	28 (45.90)	6 (28.57)	2 (10.53)
Yes	92 (29.68)	20 (37.04)	33 (54.10)	15 (71.43)	17 (89.47)

a Pearson's chi-square test for heterogeneity.

## Discussion 

Most of the respondents in this study were female, with a mean age above 40 years, and predominantly self-identified as White. These findings were also observed in other studies on the topic ^7^^-^^10^^,^^13^^-^^14^. Regarding their employment relationship with the municipal executive branch, most secretaries held appointed positions, while the majority of workers were public servants. This distinction was reflected in their length of service, with workers showing longer tenures than municipal health secretaries. 

In this regard, studies on planning and management have emphasized the importance of appointing municipal health secretaries based primarily on technical criteria rather than political ones ^7^^-^^10^, in order to ensure continuity in the planning and management processes of the Brazilian National Health System (SUS).

The two most common fields of academic training, for both categories of respondents, were nursing and administration, which is consistent with findings from other studies on the subject ^15^^-^^16^. In the present study, the third most common field was law, which may indicate a change in the profile of these agents. 

A study conducted in the state of Paraná with small-sized municipalities found that 40.3% of the municipal health secretaries interviewed did not have a university degree ^15^. In the present study, this figure was 37.47%. These findings highlight the need to change the profile of SUS managers, aiming for higher qualifications and university education ^13^. 

In this study, 37.05% of municipal health secretaries reported having previous experience in SUS planning before taking on their current role, which aligns with findings from the National Survey of Municipal Health Secretaries ^14^. Furthermore, only 88% of municipal health secretaries stated that they were responsible for managing the Municipal Health Fund, a finding that is inconsistent with the provisions in item III of Article 9, combined with paragraph 2 of Article 32 of Law No. 8,080 of September 19, 1990 ^1^.

The findings show that municipalities with larger populations were more likely to have staff working exclusively in SUS planning, a dedicated planning department, and personnel formally employed by the municipal health department responsible for entering data into the Brazilian Health Budget Information System. This finding is relevant, as it aligns with the need for trained professionals specifically dedicated to this area ^17^, helping to prevent outsourcing processes ^18^ and the dismantling of the public health system ^19^.

As limitations of this study, it is important to highlight the difficulty faced by managers and workers in reporting on their work processes, particularly due to concerns about corrective or punitive repercussions. Additionally, there is a limited body of quantitative academic literature on the subject, which prevents a more in-depth analysis of how this agenda is structured at both the regional and national levels.

Finally, considering the complexity of health planning and management processes, the contribution of this study may help better understand the profile of municipal health secretaries and workers operating in this field. Moreover, understanding who these agents are may support public policy analyses, research agendas, and the adoption of strategies to enhance planning and management within the Brazilian National Health System.

## Data Availability

The data used in this study are under the custody of the authors and have not been deposited in a public repository, as this is the first article derived from the research. The data may be made available upon reasonable request, taking into account the applicable ethical and legal restrictions. Interested parties should contact the corresponding author.

## References

[1] 1. Brasil. Presidência da República. Casa Civil. Subchefia para Assuntos Jurídicos. Lei nº 8.080, de 19 de setembro de 1990. Dispõe sobre as condições para a promoção, proteção e recuperação da saúde, a organização e o funcionamento dos serviços correspondentes e dá outras providências. Diário Oficial da União. 1990 Mar 30 [cited 2025 Mar 15]. Available from: Available from: https://www.planalto.gov.br/ccivil_03/leis/l8080.html .

[2] 2. Berretta IQ, Lacerda JT, Calvo MCM. Modelo de avaliação da gestão municipal para o planejamento em saúde. Cad Saúde Pública. 2011;27(11):2143-54. 10.1590/s0102-311x2011001100008 22124492

[3] 3. Rivera FJU, Artmann E. Planejamento e gestão em saúde: histórico e tendências com base numa visão comunicativa. Ciênc saúde coletiva. 2010;15(5):2265-74. 10.1590/s1413-81232010000500002 20802860

[4] 4. Vieira FS. Avanços e desafios do planejamento no Sistema Único de Saúde. Ciênc saúde coletiva. 2009;14(supl.1):1565-77. 10.1590/s1413-81232009000800030 19750366

[5] 5. Lacerda JT, Calvo MCM, Berretta IQ, Ortiga AMB. Avaliação da gestão para o planejamento em saúde em municípios catarinenses. Ciênc saúde coletiva. 2012;17(4):851-9. 10.1590/s1413-81232012000400008 22534840

[6] 6. Fuginami CN, Colussi CF, Ortiga AMB. Análise dos instrumentos de gestão elaborados pelas Secretarias Municipais de Saúde de Santa Catarina no período de 2014 a 2017. Saúde Debate. 2020;44(126):857-70.

[7] 7. Arcari JM, Barros APD, Rosa RS, Marchi RD, Martins AB. Perfil do gestor e práticas de gestão municipal no Sistema Único de Saúde (SUS) de acordo com porte populacional nos municípios do estado do Rio Grande do Sul. Ciênc saúde coletiva. 2020;25(2):407-20. 10.1590/1413-81232020252.13092018 32022182

[8] 8. Ouverney ALM, Carvalho ALB, Machado NMS, Moreira MR, Ribeiro JM. Gestores municipais do Sistema Único de Saúde: perfil e perspectivas para o Ciclo de Gestão 2017-2020. Saude Debate. 2019;43(spe7):75-91.

[9] 9. Riquieri MRL, Carvalho ALB, Ouverney ALM, Sarti TD. Perfil dos secretários municipais de Saúde do Brasil: um panorama de três décadas. Rev Adm Pública. 2022;56(5):683-93.

[10] 10. Aguiar VR, Cabreira FS, Ritter F, Celeste RK. Quais aspectos influenciam a priorização da Atenção Primária à Saúde pelos gestores municipais do Rio Grande do Sul - Brasil? Ciênc saúde coletiva. 2023;28(1):197-208. 10.1590/1413-81232023281.00182022 36629564

[11] 11. Instituto Brasileiro de Geografia e Estatística. Cidades e Estados [Internet]. [cited 2025 Abr 3]. Available from: Available from: https://www.ibge.gov.br/cidades-e-estados/rs/ .

[12] 12. Rio Grande do Sul. Secretaria da Saúde. Plano Estadual de Saúde: 2024-2027 [internet]. Organizado pelo Grupo de Trabalho Planejamento, Monitoramento e Avaliação da Gestão. Porto Alegre: ESP/SES; 2023. 348p [cited 2025 Abr 10]. Available from: Available from: https://saude.rs.gov.br/upload/arquivos/202410/30121947-plano-estadual-saude-versao-final-site.pdf .

[14] 14. Fleury S, Ouverney ALM. The new profile of local managers in the decentralized health system in Brazil. Saúde Debate. 2018;42(119):809-25.

[15] 15. Carvalho ALB, Ouverney ALM, Carvalho MGO, Machado NMS. Enfermeiros gestores no Sistema Único de Saúde: perfil e perspectivas com ênfase no Ciclo de Gestão 2017-2020. Ciênc saúde coletiva. 2020;25(1):211-22. 10.1590/1413-81232020251.29312019 31859869

[16] 16. Pinafo E, Carvalho BG, Almeida EDFP, Domingos CM, Bonfim MCB. O gestor do sus em município de pequeno porte: perfil, funções e conhecimento sobre os instrumentos de gestão. Espaço para Saúde. 2016;17(1):130-7.

[17] 17. Ohira RHF, Cordoni Junior L, Nunes EFPA. Perfil dos gerentes de Atenção Primária à Saúde de municípios de pequeno porte do norte do Paraná, Brasil. Ciênc saúde coletiva. 2014;19(2):393-400. 10.1590/1413-81232014192.21952012 24863816

[18] 18. Batista KBC, Gonçalves OSJ. Formação dos profissionais de saúde para o SUS: significado e cuidado. Saude soc. 2011;20(4):884-99.

[19] 19. Silva KSB, Bezerra AFB, Sousa IMC, Gonçalves RF. Conhecimento e uso do Sistema de Informações sobre Orçamentos Públicos em Saúde (SIOPS) pelos gestores municipais, Pernambuco, Brasil. Cad Saúde Pública. 2010;26(2):373-82. 10.1590/s0102-311x2010000200016 20396852

[20] 20. Mendes Á, Carnut L, Melo M. Continuum de desmontes da saúde pública na crise do covid-19: o neofascismo de Bolsonaro. Saude soc. 2023;32(1):e210307pt.

