# Supplementary material for: Profile of the Health Planning Management Workforce: a cross-sectional study, Rio Grande do Sul, Brazil, 2023
Source: Epidemiol Serv Saude. 2026 Apr 4;35:e20250590. doi: 10.1590/S2237-96222026v35e20250590.en (PMC13686387; doi:10.1590/S2237-96222026v35e20250590.en)
Supplement: Translation

# Translation: Perfil da força de trabalho da gestão do planejamento em saúde: estudo transversal, Rio Grande do Sul, 2023

**DOI:** 10.1590/S2237-96222026v35e20250590.pt

**Authors:** Caroline Cardozo Bortolotto, Pedro San Martin Soares, André Luis Alves de Quevedo, Giuliani Schwantz, Isana Oliveira da Silva, Cristian Fabiano Guimaraes

**Affiliations:** 1 Secretaria da Saúde do Estado do Rio Grande do Sul, Porto Alegre, RS, Brazil Secretaria da Saúde do Estado do Rio Grande do Sul Porto Alegre RS Brazil; 2 Tribunal de Contas do Estado do Rio Grande do Sul, Porto Alegre, RS, Brazil Tribunal de Contas do Estado do Rio Grande do Sul Porto Alegre RS Brazil; 3 Universidade Federal de São Paulo, Departamento de Medicina Preventiva da Escola Paulista de Medicina, São Paulo, SP, Brazil Universidade Federal de São Paulo, Departamento de Medicina Preventiva da Escola Paulista de Medicina São Paulo SP Brazil

**Keywords:** Planejamento em Saúde, Pessoal de Saúde, Gestor de Saúde, Gestão em Saúde, Serviços de Saúde

## Abstract

**Objetivo:**

Descrever o perfil da força de trabalho na gestão do planejamento do Sistema Único de Saúde (SUS) no âmbito das secretarias municipais de saúde do Rio Grande do Sul, Brasil.

**Métodos:**

Estudo transversal com secretários e trabalhadores municipais da gestão e do planejamento da saúde por meio de questionários online autoaplicáveis, executado entre setembro e novembro de 2023. Foi realizada análise descritiva e associações, considerando significância estatística para p-valor<0,05 pelo teste chi-quadrado de Pearson*.*

**Resultados:**

Dos 497 municípios, participaram do estudo 467 secretários de saúde e 431 trabalhadores municipais da gestão do planejamento do SUS. A maioria era do sexo feminino, com média de idade acima de 40 anos e raça/ cor da pele branca. Os trabalhadores referiram uma maior escolaridade do que os secretários municipais de saúde. Quanto ao vínculo com o poder executivo municipal, a maioria dos secretários detinham cargos comissionados (74,08%), ao passo que os trabalhadores eram estatutários (60,32%). Cerca de 68% dos trabalhadores da saúde relataram não trabalhar exclusivamente na gestão do planejamento em saúde no município. Quanto maior o porte populacional do município, maior a probabilidade de haver atuação exclusiva do trabalhador e um setor específico para a gestão do planejamento do SUS.

**Conclusão:**

Compreender quem são os atores que conduzem o planejamento e a gestão do SUS pode ajudar a subsidiar análises de políticas públicas e a adotar estratégias que qualifiquem o sistema e melhorem a situação de saúde da população.

**Ethical aspects**

Esta pesquisa respeitou os princípios éticos, obtendo os seguintes dados de aprovação:

Comitê de ética em pesquisa Escola de Saúde Pública do Rio Grande do Sul

Número do parecer: 6.277.600

Data de aprovação: 1/9/2023

Certificado de apresentação de apreciação ética: 69138023.9.0000.5312

Registro do consentimento livre e esclarecido: Obtido de todos os participantes.

## Introdução 

O planejamento governamental, na forma contemporânea, teve sua institucionalização no território brasileiro especialmente a partir de 1990. Segundo a Lei nº 8.080, de 19 de setembro de 1990 ^1^, que institui o Sistema Único de Saúde (SUS), compete à direção nacional do sistema a elaboração do planejamento estratégico nacional, em cooperação técnica com os estados, os municípios e o Distrito Federal, valorizando a lógica ascendente no processo de planejamento. Portanto, um planejamento potente depende de estrutura e de força de trabalho consistentes nos estados e nos municípios.

No presente artigo, entende-se por força de trabalho da gestão do planejamento em saúde os secretários municipais de saúde e os trabalhadores vinculados a essa área da administração pública brasileira. Aos primeiros, escolhidos pelo governo eleito democraticamente, compete especialmente a tomada de decisão dos processos de gestão; aos segundos, a estruturação e a operacionalização técnica e política das ações realizadas nesse campo.

O SUS é um sistema complexo que requer planejamento eficiente para garantir a prestação de serviços de saúde de qualidade à população. Por essa via, entende-se que o planejamento consiste em um instrumento de pactuação de responsabilidades das três esferas de governo que, além de requisito legal, permite a definição de objetivos e a organização das ações a serem desenvolvidas. Nesse contexto, a função de planejamento é expressa por meio de instrumentos e de ferramentas desenvolvidas com intencionalidade, bem como por processos de trabalho que requerem conhecimento técnico e de gestão ^2^^-^^5^.

A gestão em saúde possui inúmeros desafios com relação à tomada de decisão, sendo o planejamento uma função necessária para subsidiá-los nas articulações inerentes a esse processo complexo e dinâmico. Contudo, apesar de existir consenso de que o planejamento é dispositivo estratégico para pensar a política pública de saúde no Brasil, o planejamento em saúde pouco tem sido pensado enquanto estratégia e política, com vistas à análise das suas potências e dos seus desafios ^2^^-^^3^. 

A partir dos anos 2000, houve no SUS um forte incentivo aos processos de gestão e de planejamento em saúde no âmbito das secretarias estaduais e municipais de saúde. Por meio do Sistema de Planejamento do Sistema Único de Saúde constituiu-se uma política para indução sistemática dos instrumentos normativos de gestão, como os planos de saúde, as programações anuais de saúde e os relatórios de gestão ^4^^-^^6^, visando ao fortalecimento do exercício dos trabalhadores que atuam na área.

Mesmo com os avanços nesse campo, poucos estudos na literatura acadêmica têm avaliado o perfil dos secretários e dos trabalhadores municipais de saúde que atuam na gestão do planejamento do SUS ^2^^,^^5^^,^^7^^-^^10^, o que gera uma lacuna no conhecimento e nos processos de fortalecimento da gestão e do planejamento em saúde. Assim, o objetivo do presente artigo é descrever o perfil da força de trabalho na gestão do planejamento do Sistema Único de Saúde no âmbito das secretarias municipais de saúde do Rio Grande do Sul. 

## Métodos 

Delineamento do estudo

Trata-se de estudo transversal realizado no Rio Grande do Sul com 467 secretários municipais de saúde e 431 trabalhadores municipais da saúde responsáveis pela gestão do planejamento do Sistema Único de Saúde nos municípios do estado.

Contexto

O Rio Grande do Sul é composto por 497 municípios e uma população de 10.882.965 pessoas, conforme o censo brasileiro de 2022 ^11^. Quanto ao porte populacional, 335 municípios têm até 10 mil habitantes; 55 municípios têm entre 10 mil e 20 mil habitantes; 64 municípios entre 20 mil e 50 mil; 24 municípios possuem entre 50 mil e 100 mil; e 19 municípios acima de 100 mil habitantes ^8^. Territorialmente, a organização do sistema de saúde está agrupada em sete macrorregiões de saúde (Centro-Oeste, Metropolitana, Missioneira, Norte, Serra, Sul e Vales), 18 coordenadorias regionais de saúde e 30 regiões de saúde ^12^.

Participantes

Foram elegíveis para participar do estudo todos os indivíduos maiores de 18 anos de idade e que estavam ocupando o cargo de secretário municipal de saúde ou de trabalhador responsável pelo planejamento do Sistema Único de Saúde (SUS) na secretaria municipal de saúde dos municípios do Rio Grande do Sul. Quanto às tentativas para ampliar a adesão dos participantes, foram utilizados espaços de gestão da saúde, como as comissões intergestores regionais, grupos de trabalho institucionais, contatos via Tribunal de Contas do Rio Grande do Sul e Conselho de Secretarias Municipais de Saúde do Rio Grande do Sul, além de envio de ofício aos prefeitos municipais explicando sobre a relevância do estudo.

Variáveis

As variáveis selecionadas para o presente artigo foram: sexo (masculino; feminino), idade (em anos), raça/cor da pele (branca; preta; parda; outras), escolaridade (ensino fundamental incompleto/completo; ensino médio incompleto/completo; ensino superior incompleto; ensino superior completo; especialização; mestrado/doutorado), tipo de vínculo com o poder executivo municipal (estatutário; comissionado; celetista; contratado por prazo determinado; terceirizado/prestador de serviço), área de graduação (lista de profissões da área da saúde, das ciências humanas e das ciências sociais aplicadas, além de campo aberto), pós-graduação em gestão ou em planejamento em saúde pública (sim; não), experiência em gestão do planejamento do SUS antes de se tornar secretário municipal de saúde (sim; não), experiência prévia em gestão do planejamento do SUS (em anos), tempo de vinculação à secretaria municipal de saúde (em anos), trabalho exclusivo com gestão do planejamento do SUS no município (sim; não), existência de setor específico para trabalhar com o planejamento do SUS na secretaria municipal de saúde (sim; não), vínculo dos trabalhadores responsáveis pelo lançamento das informações no Sistema de Informações sobre Orçamentos Públicos em Saúde com a secretaria municipal de saúde (sim; não), gestor do Fundo Municipal de Saúde ser o secretário municipal de saúde (sim; não); porte populacional (<10 mil habitantes; 10 mil a <20 mil habitantes; 20 mil a <50 mil habitantes; 50 mil a <100 mil habitantes; ≥100 mil habitantes).

Fonte, mensuração de dados e controle de viés

Os dois instrumentos de coleta de dados (para secretários e para trabalhadores da gestão do planejamento do Sistema Único de Saúde [SUS] municipal) foram enviados pelo sistema interno do Tribunal de Contas do Rio Grande do Sul. Ambos eram questionários online autoaplicáveis e foram constituídos com base nas experiências prévias dos pesquisadores e em um estudo realizado em Santa Catarina, cujo objetivo foi avaliar a gestão do planejamento em saúde em municípios catarinenses ^2^.

Inicialmente, foi realizado um estudo piloto para testar os instrumentos de coleta de dados e avaliar a viabilidade e a efetividade da metodologia de estudo proposta. Para isso, foram sorteadas três macrorregiões de saúde do Rio Grande do Sul (Centro-Oeste, Missioneira e Vales) e, posteriormente, sorteados três municípios (Silveira Martins, Boa Vista do Incra e Herveira) com menos de três mil habitantes - sendo um por macrorregião de saúde selecionada. Foram realizadas análises para avaliar a consistência das respostas e a qualidade dos dados obtidos. Após esse processo, o estudo foi ampliado para os demais municípios do Rio Grande do Sul. Os municípios incluídos no estudo piloto não foram considerados na análise final do presente estudo.

Quanto ao recrutamento e à seleção de participantes, primeiramente, foi enviada por e-mail uma carta de apresentação do estudo aos secretários municipais de saúde. Através da carta de apresentação, o(a) secretário(a) municipal comunicava aos trabalhadores responsáveis pela gestão do planejamento do SUS do município sobre o estudo e o contato posterior a ser realizado para o convite de preenchimento do questionário. 

A coleta de dados ocorreu entre os meses de setembro e novembro de 2023 e foi supervisionada por dois epidemiologistas. A verificação da consistência dos dados foi realizada semanalmente. Por fim, as estratégias de controle de viés empregadas foram: a definição dos critérios de inclusão e de exclusão, a supervisão da coleta de dados, a checagem de consistência das respostas, as análises de perdas e a estratificação dos resultados.

Análise estatística

O presente estudo não utilizou cálculo de amostra, pois a pesquisa buscou contemplar o universo das secretarias municipais de saúde no formato de censo municipal da gestão do planejamento em saúde do SUS. As análises dos dados foram realizadas no pacote estatístico Stata 17.0. Inicialmente, foram realizadas análises descritivas das variáveis, sendo que as categóricas foram apresentadas em frequências absolutas e relativas. Para os dados com distribuição simétrica, foram analisadas as médias e os desvios-padrão (DP), enquanto para os dados com distribuição assimétrica, a mediana e o intervalo-interquartil (IQ). A fim de verificar associações, foi utilizado o teste chi-quadrado de Pearson, sendo considerado significativo um p-valor<0,05.

## Resultados 

Dos 497 municípios, foram obtidas respostas para 467 (93,96%) secretários municipais de saúde e 431 (86,72%) trabalhadores municipais do planejamento em saúde. A maioria dos respondentes era do sexo feminino (52,68% para secretários e 77,26% para trabalhadores). A distribuição da idade se concentrou entre 30 anos e 59 anos. Os secretários apresentaram maior prevalência de indivíduos com 60 anos ou mais em relação aos trabalhadores (12,21% e 3,48%, respectivamente). Ademais, 93,27% dos respondentes declararam ter raça/cor da pele branca (Tabela 1). A média de idade para os secretários e para os trabalhadores foi de 45,96 anos (IC95% 45,05; 46,87; DP 10,05) e de 41,28 anos (IC95% 40,45; 42,10; DP 8,70), respectivamente. 

Quanto à escolaridade, 37,47% dos secretários não possuíam ensino superior completo; 26,98% dos secretários e 43,39% dos trabalhadores tinham alguma especialização. Os secretários eram majoritariamente vinculados ao poder executivo municipal por meio de cargos comissionados (74,08%), ao passo que os trabalhadores eram, em sua maioria, estatutários (60,32%) (Tabela 1).

Sobre a área de graduação, os secretários municipais de saúde com ensino superior completo (n=292) elencaram as principais áreas: enfermagem (n=62), administração (n=46), direito (n=24), gestão em saúde (n=20), pedagogia (n=14), serviço social (n=13) e outras áreas (n=113). Por sua vez, os trabalhadores que tinham ensino superior completo (n=367) informaram as seguintes áreas: enfermagem (n=180), administração (n=33), direito (n=17), ciências contábeis (n=16), serviço social (n=8), odontologia (n=8) e outras áreas (n=105).

**Tabela 1:** Características dos secretários (n=467) e trabalhadores municipais (n=431) de saúde da gestão do planejamento do Sistema Único de Saúde. Rio Grande do Sul, 2023

Variável	Secretários n (%)	Trabalhadores n (%)
Sexo		
Feminino	246 (52,68)	333 (77,26)
Masculino	221 (47,32)	98 (22,74)
Faixa etária (anos)		
18-29	12 (2,57)	35 (8,12)
30-39	119 (25,48)	151 (35,03)
40-49	176 (37,69)	178 (41,30)
50-59	103 (22,06)	52 (12,06)
60≥	57 (12,21)	15 (3,48)
Raça/cor da pele		
Branca	437 (93,58)	402 (93,27)
Preta	25 (5,35)	19 (4,41)
Parda/outras	5 (1,07)	10 (2,31)
Escolaridade		
Ensino fundamental incompleto/completo	19 (4,07)	2 (0,46)
Ensino médio incompleto/completo	107 (22,91)	39 (9,05)
Ensino superior incompleto	49 (10,49)	23 (5,34)
Ensino superior completo	149 (31,91)	163 (37,82)
Especialização	126 (26,98)	187 (43,39)
Mestrado/doutorado	17 (3,64)	17 (3,94)
Vínculo com o poder executivo municipal		
Estatutário	108 (23,13)	260 (60,32)
Cargo comissionado	346 (74,08)	114 (26,45)
Celetista	4 (0,86)	9 (2,09)
Contratado por prazo determinado	7 (1,50)	28 (6,50)
Terceirizado/prestador de serviço	2 (0,43)	20 (4,64)

Dos 292 secretários que detinham ensino superior completo, 29,45% (n=86) responderam possuir pós-graduação em gestão ou em planejamento em saúde; para os trabalhadores, essa proporção foi de 33,24%. Sobre ter experiência prévia em gestão do planejamento do Sistema Único de Saúde (SUS), 37,05% (n=179) dos secretários municipais informaram que a possuíam antes de assumir o cargo atual, e a mediana de tempo de experiência prévia em gestão do planejamento do SUS para esses atores foi de 2,75 anos (IQ 0,58-6,67 anos).

Quanto ao tempo de vínculo com a secretaria municipal de saúde, a mediana foi de 2,75 anos (IQ 0,83-6,25 anos) para os secretários e de 4,8 anos (IQ 1,7-13 anos) para os trabalhadores. Estratificando-se o tempo de vínculo, a categoria "menos do que cinco anos" contemplou 71,73% dos secretários e 51,51% dos trabalhadores participantes do estudo. Além disso, 11,56% dos secretários e 22,97% dos trabalhadores encontravam-se na categoria maior que 10 anos a 20 anos de vínculo. Ainda, não houve significância estatística entre o tempo de vínculo e o porte populacional do município.

Cerca de 68% dos trabalhadores da saúde relataram não trabalhar exclusivamente na gestão do planejamento no âmbito municipal. Ao estratificar por porte populacional, observou-se que os municípios com menos de 10 mil habitantes tiveram 31,3 pontos percentuais a mais de respostas de indivíduos que não trabalhavam apenas com a gestão do planejamento comparado aos municípios com 100 mil habitantes ou mais (72,50% versus 41,20%; p-valor 0,027) (Tabela 2). 

Mais da metade (50,96%) dos secretários e dos trabalhadores (51,28%) informaram que há um setor específico para trabalhar com o planejamento do SUS na secretaria municipal de saúde. Além disso, municípios com 100 mil habitantes ou mais apresentaram maior prevalência de possuir um setor específico para trabalhar com o planejamento do SUS (84,21%; p-valor 0,025, para secretários; 88,24%; p-valor 0,006, para trabalhadores) (Tabela 3).

Quando perguntado aos secretários de saúde se os responsáveis pelo lançamento das informações no Sistema de Informações sobre Orçamentos Públicos em Saúde eram vinculados à secretaria municipal de saúde, apenas 177 (37,90%) responderam que sim. Constatou-se que quanto maior o porte populacional dos municípios, maior foi a prevalência de ter trabalhadores vinculados à secretaria de saúde para lançar informações no Sistema de Informações sobre Orçamentos Públicos em Saúde (p-valor<0,001) (Tabela 4). Por fim, 88,15% dos secretários municipais relataram serem gestores do Fundo Municipal de Saúde. Quando essa variável foi estratificada por porte populacional, não houve significância estatística. 

**Tabela 2:** Trabalho exclusivo na gestão do planejamento do Sistema Único de Saúde (SUS), por porte populacional, trabalhadores municipais de saúde da gestão do planejamento do SUS. Rio Grande do Sul, 2023 (n=431)

Trabalho exclusivo na gestão SUS (p-valor 0,027)^a^	<10 mil habitantes	10 mil a <20 mil habitantes	20 mil a <50 mil habitantes	50 mil a <100 mil habitantes	≥100 mil habitantes
	n (%)	n (%)	n (%)	n (%)	n (%)
Não	206 (72,50)	32 (62,75)	33 (56,90)	13 (61,90)	7 (41,20)
Sim	78 (27,50)	19 (37,25)	25 (43,10)	8 (38,10)	10 (58,80)

a Teste chi-quadrado de Pearson para heterogeneidade.

**Tabela 3:** Existência de setor específico de planejamento do Sistema Único de Saúde (SUS), por porte populacional, secretários (n=467) e trabalhadores (n=431) municipais de saúde da gestão do planejamento do SUS. Rio Grande do Sul, 2023

Setor específico de planejamento do SUS	<10 mil habitantes	10 mil a <20 mil habitantes	20 mil a <50 mil habitantes	50 mil a <100 mil habitantes	≥100 mil habitantes
	n (%)	n (%)	n (%)	n (%)	n (%)
Secretários (p-valor 0,025)^a^					
Não	161 (51,77)	29 (53,70)	28 (45,16)	8 (38,10)	3 (15,79)
Sim	150 (48,23)	25 (46,30)	34 (54,84)	13 (61,90)	16 (84,21)
Trabalhadores (p-valor 0,006)^a^					
Não	153 (53,87)	22 (43,14)	25 (43,10)	8 (38,10)	2 (11,76)
Sim	131 (46,13)	29 (56,86)	33 (56,90)	13 (61,90)	15 (88,24)

a Teste chi-quadrado de Pearson para heterogeneidade.

**Tabela 4:** Vínculo de trabalhador da saúde para preencher o Sistema de Informações sobre Orçamentos Públicos em Saúde, por porte populacional, secretários municipais de saúde da gestão do planejamento do Sistema Único de Saúde. Rio Grande do Sul, 2023 (n=467)

Vínculo trabalhador saúde (p-valor<0,001)^a^	<10 mil habitantes	10 mil a <20 mil habitantes	20 mil a <50 mil habitantes	50 mil a <100 mil habitantes	≥100 mil habitantes
	n (%)	n (%)	n (%)	n (%)	n (%)
Não	218 (70,32)	34 (62,96)	28 (45,90)	6 (28,57)	2 (10,53)
Sim	92 (29,68)	20 (37,04)	33 (54,10)	15 (71,43)	17 (89,47)

a Teste chi-quadrado de Pearson para heterogeneidade.

## Discussão 

A maioria dos respondentes da presente pesquisa era do sexo feminino, com média de idade acima de 40 anos, predominantemente da raça/cor da pele autorreferida branca. Tais achados também foram encontrados em outros estudos sobre a temática ^7^^-^^10^^,^^13^^-^^14^. Quanto ao vínculo com o poder executivo municipal, a maioria dos secretários possuía cargos comissionados, enquanto a maior parte dos trabalhadores era estatutária. Tal fato se refletiu no tempo de vínculo, uma vez que os trabalhadores apresentaram maiores tempos que os secretários municipais. 

Nessa linha, estudos na área do planejamento e da gestão têm ressaltado a importância de que a escolha dos secretários municipais de saúde seja realizada especialmente a partir de critérios técnicos, acima de critérios políticos ^7^^-^^10^, com vistas à continuidade dos processos de planejamento e de gestão do Sistema Único de Saúde (SUS).

As duas primeiras áreas de formação, de ambas as categorias de entrevistados, foram enfermagem e administração, o que está de acordo com outras pesquisas sobre a temática ^15^^-^^16^. No presente estudo, a terceira posição de formação foi a jurídica, o que pode demonstrar uma mudança no perfil desses atores. 

Estudo realizado no Paraná com municípios de pequeno porte identificou que 40,3% dos secretários municipais de saúde entrevistados não possuíam graduação ^15^. Já nos dados desta pesquisa, esse valor foi de 37,47%. Tais achados sinalizam a necessidade de modificação no perfil dos gestores do SUS, no sentido de ter maiores qualificação e formação universitária ^13^. 

Neste estudo, 37,05% dos secretários municipais de saúde responderam que possuíam experiência prévia em planejamento do SUS antes de assumir o cargo atual, o que condiz com os achados da Pesquisa Nacional dos Secretários Municipais de Saúde ^14^. Ademais, apenas 88% dos secretários municipais de saúde responderam ser gestores do Fundo Municipal de Saúde, o que está em desacordo com o entendimento constante no inciso III do art. 9º, combinado com o § 2º do art. 32, da Lei nº 8.080, de 19 de setembro de 1990 ^1^.

Foi possível identificar que quanto maior o porte populacional do município, maior o resultado de haver atuação exclusiva do trabalhador e setor específico para a gestão do planejamento do SUS, bem como trabalhador com vínculo com a secretaria municipal de saúde para o preenchimento do Sistema de Informações sobre Orçamentos Públicos em Saúde. Tal achado é importante, pois vai ao encontro da necessidade de ter profissionais específicos nessa área e com formação adequada ^17^, a fim de evitar processos de terceirização ^18^ e desmantelamento do sistema público de saúde ^19^.

Como limitações desta investigação, ressaltam-se as dificuldades dos gestores e dos trabalhadores em retratar seus processos de trabalho, especialmente por receio de perspectivas corretivas e punitivas. Além disso, existe uma baixa produção acadêmica quantitativa sobre o tema em discussão, o que não permite uma análise aprofundada da organização dessa agenda em nível regional e em nacional.

Por fim, levando-se em consideração a complexidade dos processos de gestão e de planejamento em saúde, a contribuição do presente estudo pode ajudar a compreender o perfil dos secretários e dos trabalhadores municipais de saúde que atuam nessa área. Ademais, conhecer esses atores pode ajudar a subsidiar análises de políticas públicas, agendas de pesquisa e adoção de estratégias que qualifiquem o planejamento e a gestão no Sistema Único de Saúde.

## Data Availability

Os dados utilizados neste estudo estão sob a guarda dos autores e não foram depositados em um reposi­tório público, visto que este é o primeiro artigo derivado da pesquisa. Os dados podem ser disponibilizados mediante solicitação razoável, considerando as restrições éticas e legais aplicáveis. Interessados podem entrar em contato com o autor correspondente.
